# Prognostic value of D-dimer in treatment and follow up for patients who underwent radiotherapy in advanced lung cancer

**DOI:** 10.1371/journal.pone.0333085

**Published:** 2025-09-23

**Authors:** Huriye Senay Kiziltan, Kimia Cepni

**Affiliations:** Department of Radiation Oncology, University of Health Sciences, Başakşehir Çam and Sakura City Hospital, Istanbul, Türkiye; Niloufer Hospital, Institute of Child Health, INDIA

## Abstract

**Background:**

Lung carcinma, a serious disease commonly recognized at advanced stages and has less favorable outcomes to treatments. This research examines the association between D-dimer values and clinical variables in patients diagnosed with advanced lung cancer, with a focus on their prognostic significance and radiotherapy related outcomes.

**Methods:**

This retrospective observational descriptive cohort study, including 142 patients with lung cancer. Aa comprehensive analysis, was conducted, including demographic variables,clinical staging, pathology, laboratuary tests, and D-dimer levels measured both before and after the administration of radiotherapy.

**Results:**

The findings indicated that high pre-RT D-dimer concentrations were significantly assoccciated with advanced stages of disease, worse response of radiotherapy, Eastern Cooperative Oncology Group performance status, and decreased survival outcomes. Radiotherapy response also affects to survival rates with D-dimer values. The findings from both univariate and multivariable survival analyses demonstrated that lower concentrations of D-dimer correlated with better response of radiotherapy, extended overall survival and progression free survival (p: < 0.001).

**Conclusion:**

These results illustrate the significant role of D-dimer as a valuable biomarker for assessing tumor burden and guiding radiotherapy and other treatment strategies in lung cancer, thereby necessitating further inquiry to evaluate its clinical implications.

## Introduction

Lung cancer (LC) constitutes the most widespread and fatal neoplasm on the world. Diagnostic evaluations illustrate that 80% of patients diagnosed with LC manifest of advanced stage disease, correlating with a 5-year survival rate below 15%, thus a critical risk to public health [1,2]. The principal pathological variants of LC are non-small cell lung cancer (NSCLC) and small cell lung cancer (SCLC) [2]. Investigations and scholarly research have demonstrated that neoplasias can exert an impact on the hemostatic system. The stimulation of the hemostatic system may also concurrently affect the biological characteristics of neoplasms [3]. The triggering of clot formation and the dissolution of fibrin are commonly associated to several mechanism of cancer growths; yet, the detailed molecular pathways that account for this relationship are not yet well determined [4]. The manifestation of hypercoagulability noted in individuals which caused coagulation and fibrinolytic pathway altering with cancer related a sophisticated and complex biochemical and molecular changes [5].

The D-dimer defined a range of peptide fragments generated by plasmin acting on cross-linked fibrin. These elements, representing the activation of clotting and fibrin fragmentation systems [6]. The activity of thrombin sets off the change of fibrinogen to fibrin monomers, which indicates the beginning of D-dimer creation. These monomers combine to generate a fibrous matrix, which is subsequently linked by Factor XIIIa, ultimately leading to the establishment of flexible fibrin clots [7].

High D-dimer concentrations are important for assessing to thrombotic risk and also significant marker for tumor burden and advanced disease. Research evidence supports the concept that high plasma D-dimer levels are significantly related to the advanced stages of multiple neoplasms, especially lung and colorectal tumors. This discovery implies that concentrations of D-dimer could function as significant prognostic markers in the assessment of patient outcomes [8].

Cancer cells exhibite extreme blood vessel formation and ability to spread might release increased amounts of tissue factor, promote the release of clotting agents, and interact with blood vessel lining cells, platelets, and immune cells. These interactions are crucial in creating a condition of high level coagulation [9]. Evidence suggests that in a state of high level coagulability, fibrinogen, platelets, and cancer cells frequently aggregate and develop microthrombi. By means of this process, tumor cells may successfully excapes the immune surveillance and promote the phenomenon of metastasis [10].

Elevated concentrations of plasma D-dimer demonstrated to diminished survival. Analytical results demonstrate that raised D-dimer levels are linked not only to progressed disorder but also to a lesser effectiveness of cancer therapies [11].

Altough many research has showed correlation between increased D-dimer concentrations and the prognostic outcomes in LC, the predictive significance of D-dimer levels in the LC is still not clear enough. Elevated D-dimer concentrations are indicative of increased fibrinolytic activity, which is often observed in patients with malignancies including LC. This hypercoagulable state is not only a consequence of tumor growth but also plays a role in tumor progression and metastasis, as cancer cells can promote coagulation and evade immune surveillance [7,8].

Malignant cells in NSCLC might enhance the concentrations of plasma D-dimer levels via diverse physiological processes. Firstly, neoplastic cells secret the tissue factor (TF) which harms to endothelial cells and consequently boosts to thrombin formation/thereby disturbing the balance between coagulation and fibrinolysis. This phenomenon culminates in a state of hypercoagulability and the formation of thrombi in NSCLC [12]. Secondly, LC tissue exhibits a pronounced pro-inflammatory response as it invades adjacent tissues, thereby stimulating the complement system [13]. In addition, when the immune response is directed at tumor cells, the liberation of agents that encourage coagulation considerably boosts the hypercoagulable condition, thus upsetting the harmony between clotting and fibrinolysis. This augmented fibrinolytic function results in a significant elevation of D-dimer concentrations. [14].

While cytotoxic effects are obtained locally in the tumor with radiotherapy (RT), abscopal responses can also be obtained in regions distant from the tumor with immunological effects. These are the positive systemic effects of radiation. In contrast, suppression of the immune system may be worsen to response of RT. Furthermore microthrombies may increase in patients undergoing radiotherapy due to damage to the vascular structures especially endothelium of vessels of the tumor. Cytotoxic effects are obtained may increase to hypoxia and metastases [15]. Therefore, despite the tumor shrinking due to cytotoxic effects during RT, the general condition of the patient may be worsen. This is probably most important reasons why the desired maximum effect of RT is not always achieved.

Nonetheless, the application of plasma D-dimer levels as a predictive parameter for LC continues to be a controversial topic within the academic discourse. In this study we evaluated to relationship between D-dimer levels and tumor stages, disease progression, prognostic significance in forecasting outcomes, and the efficacy of RT.

There are inhomogeneous studies in the literature regarding the prognostic features of D-dimer. In this study, we aimed to show that the D-dimer values can indicate to disease sttaus also be used to guide planning RT in LC.

## Materials and methods

### Study design and participants

This retrospective observational descriptive cohort study involved 142 patients with LC between 2020 and 2022. Individuals were examined in accordance with demographic attributes, encompassing age, clinical stage, Eastern Cooperative Oncology Group (ECOG) performance status, pathological findings, and pre and post-RT D-dimer concentrations. They were also evaluated based on the characteristics of RT treatment such as RT dose, treatment regions, and response rates. Cut-off analyses were conducted on three distinct D-dimer values.

#### Inclusion criteria.

Individuals who are 18 years and older, diagnosed with LC including non-small and small cell types, whose pre and post RT of D-dimer values were measured and who have had RT.

#### Exclusion criteria.

Individuals who had received any directed molecular or immunotherapy interventions, coagulative disorders, alongside those dealing with heart failure, heart attacks, significant breathing difficulties, severe infection and medical situations that require intensive care unit attention, were not part of the study.

### Patient demographics and clinical characteristics

A total of 142 patients were included in the analysis. The study population had a median age of 59 years (range: 18–89), The majority of patients (80.98%, n = 115) were diagnosed with NSCLC. Most of the patients (73.04%, n = 105) presented with stage IV disease. Bone metastases were most prevalent (45.07%, n = 64) regarding to metastatic distribution. A significant proportion of patients (85.91%, n = 122) had an ECOG performance status greater than 2. Patient characteristics shown to Table 1.

**Table 1:** Characteristics of LC patients.

Characteristics	Patient number (n = 142)	%
Age		
18-55	62	43.66
56-74	67	47.18
75-89	13	9.15
Sex		
Female	20	14.08
Male	122	85.91
Histological type		
NSCLC	115	80.98
SCLC	27	19.02
Metastasis location		
Bone	64	45.07
Brain	32	22.53
Liver	9	6.33
No metastasis	37	26.05
Stage		
I/II	8	5.63
III	29	20.42
IV	105	73.94
ECOG		
1-2	20	14.08
3-4	122	85.94
RT location		
Lung	49	34.50
Bone	64	45.07
Brain	32	22.53
Liver	1	0.7
CT used	38	26.76

CT: Chemotherapy.

According to immunohistochemical and genetic examinations, 72 of NSCLC patıents had adenocarcinoma and 43 had squamous cell cancer.

**Fig 1:**
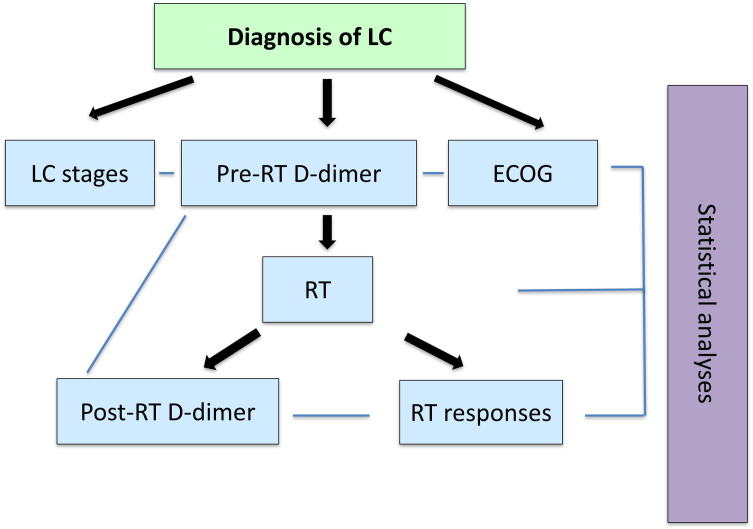
Flowchart of this study.

Fowchart of this study shown in Fig 1.

### Radiotherapy

38 patıents previosly have received CT before RT. CT was not performed during RT.

#### Curative or postoperative RT.

37 patients received curative or postoperative RT. Total RT dose was 40–66 Gy. Median RT dose per fraction was 2 Gy. In patients who underwent 4-D simulation, fusion was performed with PET CT images and internal target volume (ITV) was created after contouring gross tumor volume (GTV). After creating ITV, tumor area and involved lymph nodes were contoured by giving 0.5–1 cm margin radially and 0.7–1.5 cm margin longitudinally to create the planned target volume (PTV). Target volume coverage dose of PTV were assessed 90–95% in physical plan. Median PTV volume is 125 cc (Minimum 75, maximum 230 cc). RT was given 5 days a week for 4–7 weeks (Tables 1,2).

**Table 2:** RT characteristics and D-dimer, OS and PFS of patients.

RT characteristics	Med(Min-max)	Med D-dimer	Med OS (Months)	Med PFS (Months)
**RT dose, Gy**	1.5 (1.5–9)			
Curative/ART (n = 37, 26.06%)	2.5 (1.5–9)	0.5	17.5	15
Total dose (Gy)	60 (40–66)			
Fract. dose (Gy)	2 (1.5–2)			
Fract. number	30 (20–33)			
Palliative RT (n = 105, 73.94%)	2.5 (2.5–9)	1.5	12	10
Total dose (Gy)	30 (16–30)			
Fract. Dose (Gy)	2.5 (2–9)			
Fract. number	12 (2 –15 )			
**RT method**				
Helical Arc RT (n = 47, 33.09%)		1.1	16	14
VMAT/IMRT (n = 95, 66.01%)		1.3	12	10
**RT response rate**				
CR (n = 22, 15.49%)	100	0.2	36	36
Good (n = 14, 9.85%)	80 (70–90)	0.2	34	27
Partial (n = 93, 65.49%)	50 (40–60)	1.5 (0.2–15)	12	10
Poor (n = 13, 9.15%)	20 (−10–30)	2.1 (0.3–24)	3	0.5

Med: Median Min-max: Minimum- maximum n: Number of patient RT: Radiotherapy ART: Adjuvant radiotherapy Gy: Gray Fract: Fraction VMAT: Volumetric modulation arc radiotherapy IMRT: Intensive modulation radiotherapy. CR: Complete response PR: Partial response Poor: Stationary or progression.

#### Palliative RT.

Palliative RT was given to 105 patients. It was applied with a 0–5 mm margin according to the location. 90–95% isodose was planned to cover the PTV. Median PTV volume is 116 cc (Minimum 8, maximum 755 cc). RT total dose is 16–30 Gy. Median RT per ftaction dose is 2.5 Gy. RT was applied 1–5 days a week for 1–3 weeks.

IMRT is a radiation method which delivery of radiation dose in multiple small volumes to conform more precisely to accordance shape of the tumor by modulating the intensity of the beams applied to patients with Linear Accelerator (LINAC). VMAT arc therapy used which planned with different izocenters to provides effective treatment and decreased side effects of radiation in patients especially have large volume of tumors applied to patients with Linear Accelerator (LINAC). IMRT and VMAT applied with LINAC based teletherapy machines (Elekta Versa HD, Stocholm, Sweeden, 2020 or Tomotherapy Radixact Accuray, X7, 2020). In Helical Arc RT, the gantry which includes radiation source in Tomotherapy machine rotates around the patient in a spiral pattern during Helical arc radiotherapy. Tomotherapy (Tomotherapy Radixact Accuray, X7, 2020) is a radiation delivery machine used with 3-dimensional (3-D) image guided of radiation. Furthermore it is a high-tech type of IMRT [16] (Tables 1,2).

### Statistical analysis

All statistical analyses were performed using R software (version 4.2.0). The normality of variables was assessed using Kolmogorov-Smirnov and Shapiro-Wilk tests, along with Q-Q plots and histograms. Continuous variables were expressed as median (minimum-maximum) and mean ± standard deviation, while categorical variables were presented as frequencies (percentages). Mann-Whitney U and Kruskal-Wallis tests were used for independent continuous variables, and Wilcoxon signed-rank test for dependent variables. Relationships between continuous variables were examined using Spearman’s correlation test. Log-rank Kaplan-Meier analysis was performed to compare OS and PFS rates. ROC and DCA curve was also created for D-dimer cut-off values and survival levels. Factors affecting recurrence risk were evaluated using univariate Cox regression analysis, and variables significant in univariate analysis were included in multivariate Cox regression analysis. Model fits were assessed using likelihood ratio, and concordance values. P < 0.05 was considered statistically significant.

#### Ethical approve.

Ethical approvel was received from the Ethics Committee of Başakşehir Çam and Sakura City Hospital (Date of Ethical approve is 05.2023, No:177). The authors began reviewing patient data on 01.06.2023.

#### Informed consents.

No human subjects were studied in this study. Since it was a retrospective study in which medical records and archived samples were evaluated and analyzed, Ethics Committee approval was received, but patient informed consent was not required by the ethics committee because this is a non interventional study.

## Results

### RT treatment parameters and initial outcomes

The median daily RT dose was 250 cGy with a median fraction number of 12 with a median total RT dose of 30 Gy (Table 2).

### Hematological and biochemical parameters

Post-RT laboratory evaluations revealed comprehensive insights into patients hematological and inflammatory status. The median hematocrit was 36.0% (range: 12.2–49.3%), with a mean value of 35.0 ± 6.1%. Inflammatory markers demonstrated considerable variation among patients: CRP levels had a median of 30 mg/dL (range: 1–214 mg/dL), and ferritin levels showed a median of 392 ng/mL (range: 14–2,674 ng/mL). Coagulation parameters were also assessed, with fibrinogen showing a median of 370 mg/dL (range: 54–915 mg/dL) and INR maintaining a median of 1.00 (range: 0.80–2.20). These laboratory parameters provided valuable insights into the patients’ systemic response to treatment and overall disease status.

### D-dimer and relation with clinical parameters

The median D-dimer level before RT was 1.10 µg/mL (range: 0.10–15.00), which showed a slight decrease to 0.80 µg/mL (range: 0.10–20.00) after RT, although this difference did not reach statistical significance (p = 0.531). Pre-RT D-dimer levels demonstrated significant associations with several clinical parameters: patients with stage IV disease (p = 0.007), ECOG performance status >2 (p = 0.003), and those who did not survive during follow-up (p = 0.002) had significantly elevated levels. Similar patterns persisted in post-RT D-dimer levels, maintaining significant associations with metastasis location (p = 0.026), ECOG status (p = 0.018), and survival status (p < 0.001) (Table 3).

**Table 3:** Pre-RT and post RT D-dimer levels in associated with prognostic significance.

Parameters	Pre- RT		Post-RT	
	D-dimer, µg/mL^1^	p-value^2^	D-dimer, (µg/mL^1^)	p-value^2^
**Histological type**		0.728		0.492
NSCLC	1.33 (0.10–4.30)		0.80 (0.10–20.00)	
SCLC	0.61 (0.25–15.00)		1.00 (0.20–8.51)	
**Metastasis location**		**<0.001**		**0.026**
Bone	1.55 (0.33–15.00)		1.95 (0.36–8.51)	
Brain	1.33 (0.10–11.00)		1.50 (0.10–20.00)	
Liver	0.40 (0.20–4.20)		0.70 (0.40–3.00)	
None	0.50 (0.10–1.90)		0.50 (0.10–4.51)	
**Stage**		**0.007**		0.086
I/II/III	0.50 (0.10–1.90)		0.50 (0.10–20.00)	
IV	1.50 (0.10–15.00)		1.00 (0.10–8.51)	
**ECOG**		**0.003**		**0.018**
≤2	0.50 (0.10–1.90)		0.50 (0.10–20.00)	
>2	1.50 (0.10–15.00)		1.20 (0.10–8.51)	
**RT location**		**<0.001**		0.111
Lung	0.56 (0.10–1.94)		0.70 (0.10–4.51)	
Bone	1.50 (0.30–15.00)		1.95 (0.30–8.51)	
Brain	0.60 (0.10–11.00)		1.05 (0.10–20.00)	
Liver	4.20 (4.20–4.20)		3.00 (3.00–3.00)	
**CT**		0.108		**0.031**
No	1.50 (0.20–15.00)		1.50 (0.20–7.83)	
Yes	0.90 (0.10–2.78)		0.68 (0.10–20.00)	

^1^Median (Min-Max)

^2^Mann-Whitney U test; Kruskal-Wallis test

Note: Significant p-values are shown in bold.

The effect of pre-RT D-dimer on post-RT changes in D-dimer levels and on survival rates are presented in Table 4.

**Table 4:** The effect of pre-RT D-dimer levels on post-RT changes in D-dimer levels and on survival rates.

Pre RT D-dimer (µg/mL)	Median total RT Dose (Gy)	Median. Post RT + / - D-dimer (µg/mL)	n (%)	Median OS (Months)	Med.ian PFS (Months)
0.1-1	60	−0.15	71 (50)	17.5	15
1.1-2	30	0.4	36 (25.35)	12	10
2.1-3	30	1.1	23 (16.19)	6	3
3.1-24	30	0.8	12 (8.45)	3	0.5

n: Patient number

In 71 patients (50%), the pre-RT D-dimer value was below 1 µg/mL, with a median decrease of 0.15 µg/mL observed after RT. In 36 patients (25.35%) with D-dimer values 1.1–2 µg/mL, an increase in D-dimer values was observed median increaase of 0.4/mL was observed after RT. In 23 patients (16.19%) with D-dimer values 2.1–3 µg/mL, a median increase of 1.1 µg/mL was observed in D-dimer values after RT. These findings were not found to be statistically significant.

For pre-RT D-dimer levels were found to be significantly higher in patients with bone metastasis in stage IV disease compared to other metastatic sites (p < 0.001). They were also significantly higher in patients with stage IV disease compared to other stages (p < 0.007)..Conversely, pre-RT D-dimer levels were found to be significantly lower in patients with ECOG ≤2 compared to those with ECOG >2. (p < 0.003). For pre-RT D-dimer levels were found to be significantly lower in patients who underwent lung region RT compared to those who received RT to other sites (p < 0.001).

### Correlations of inflammatory and laboratory parameters

Detailed correlation analyses revealed significant correlations relationships between D-dimer levels and various laboratory parameters. Moderate positive correlations were observed with inflammatory markers: CRP (r = 0.58, p < 0.001), IL-6 (r = 0.61, p < 0.001) and ferritin (r = 0.63, p < 0.001). Fibrinogen and IL-6 also showed a moderate positive correlation (r = 0.51, p < 0.001 and r = 0.61, p < 0.001). Notably, a moderate negative correlation was found with lymphocyte percentage (r = −0.63, p < 0.001), RT response (r = −0.50, p < 0.001), while weak negative correlations were observed with hematocrit (r = −0.33, p = 0.005), and INR (r = −0.48, p < 0.001).

### Survival analysis based on D-dimer thresholds

**Fig 2:**
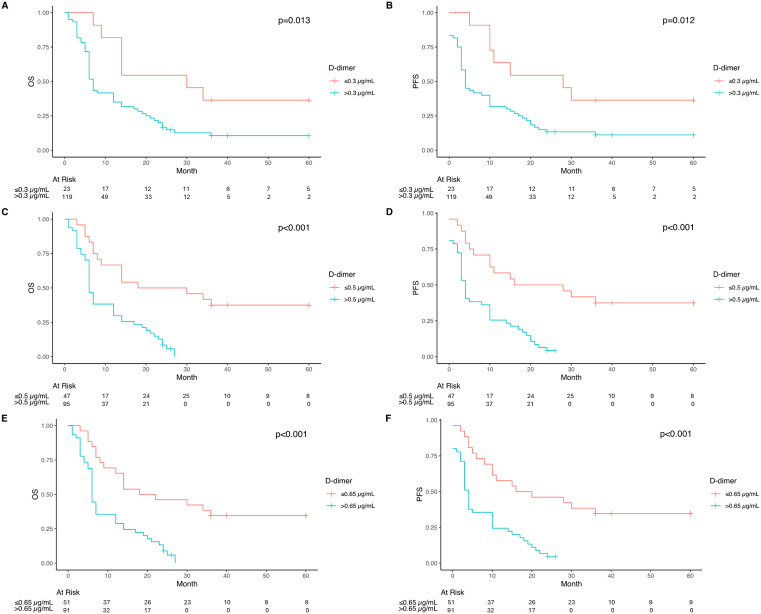
Kaplan Meier curves of OS and PFS according to d-dimer values comparing OS rates between patients with D-dimer levels ≤ 0.3 µg/mL and those with levels > 0.3 µg/mL. A. Comparing OS rates between patients with D-dimer levels ≤ 0.3 µg/mL and those with levels > 0.3 µg/mL.; B. Comparing PFS rates between patients with D-dimer levels ≤ 0.3 µg/mL and those with levels > 0.3 µg/mL. C. Comparing OS rates between patients with D-dimer levels ≤0.5 µg/ml and those with levels >0.5 µg/ml.; D. Comparing PFS rates between patients with D-dimer levels ≤0.5 µg/ml and those with levels >0.5 µg/ml.; E. Comparing OS rates between patients with D-dimer levels ≤0.65 µg/ml and those with levels >0.65 µg/ml; F. Comparing PFS rates between patients with D-dimer levels ≤0.65 µg/ml and those with levels >0.65 µg/ml.

hree different D-dimer cut-off values were determined, with univariate, multivariable and ROC analyses were performed to more precisely evaluate the relationship between D-dimer levels and survival rates in this study. Using a cut-off of ≤0.3 µg/mL, patients with lower D-dimer levels demonstrated significantly longer median OS (30 months, 95% CI: 14-NA) compared to those with >0.3 µg/mL levels (7 months, 95% CI: 6–12, p = 0.013). This survival advantage was consistently observed decreasing with higher cut-off values: at 0.5 µg/mL (24 vs 6 months, p < 0.001) and 0.65 µg/mL (20 vs 5 months, p < 0.001). PFS analysis revealed similar patterns, with patients having lower D-dimer levels consistently showing better outcomes across all cut-off values (30 vs 4 months for 0.3 µg/mL, p = 0.012; 22 vs 4 months for 0.5 µg/mL, p < 0.001; 18 vs 3 months for 0.65 µg/mL, p < 0.001) (Fig 2).

When patients were stratified into five categories based on pre-RT D-dimer levels, significant differences in survival outcomes were observed (p < 0.001). For OS, patients with D-dimer levels ≤0.3 µg/mL had a median survival of 30 months (95% CI: 9-NA), while those with levels between 0.1−1 µg/mL showed a median survival of 17.5 months (95% CI: 12−36). Survival times progressively decreased with higher D-dimer levels: 12 months (95% CI: 6−24) for 1.1−2 µg/mL, 6 months (95% CI: 5-NA) for 2.1−3 µg/mL, and 3 months (95% CI: 3-NA) for >3 µg/mL. Pairwise comparisons revealed particularly significant survival differences between patients with D-dimer levels >2 µg/mL compared to those with lower levels (p < 0.001). Median OS was 16 months in stage IV LC patients with D-dimer values between 0.1–0.3 µg/mL.

**Fig 3:**
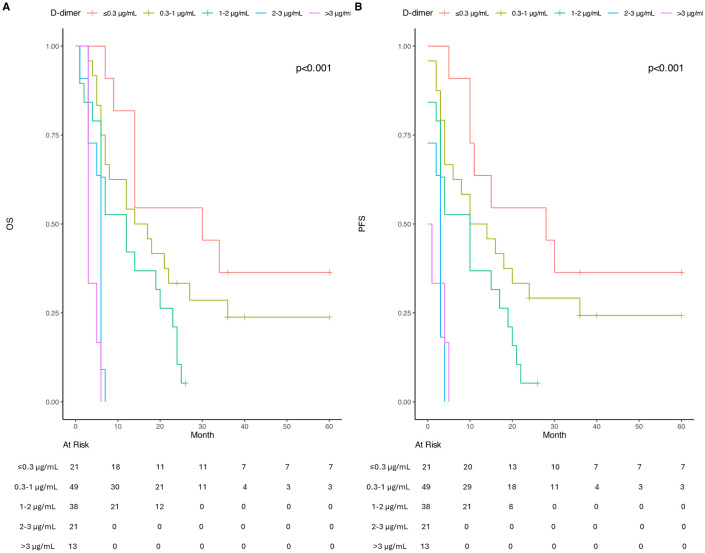
Kaplan Meier curves of OS and PFS according to different pre-RT different D-dimer values. A. Comparing OS rates for different pre-RT d-dimer values; B. Comparing PFS rates for different pre-RT d-dimer values.

Similarly, PFS demonstrated a comparable pattern, with median PFS times of 15 months (95% CI: 10-NA) for ≤0.3 µg/mL, 15 months (95% CI: 8–36) for 0.1–1 µg/mL, 10 months (95% CI: 3–20) for 1.1–2 µg/mL, 3 months (95% CI: 2-NA) for 2.1–3 µg/mL, and 0.5 months (95% CI: 0-NA) for >3 µg/mL. The most pronounced differences in both OS and PFS were observed between patients with D-dimer levels >2 µg/mL and those with lower levels (p < 0.001) (Fig 3).

### Survival based on RT response

A significant association was found between RT response and both OS and PFS. Patients with a complete response to RT (22 of patients, 15.49%) had the longest median OS at 36 months, with survival not reached within the follow-up period (NA, 95% CI: 34-NA). Those with a good partial response (70–90% respomse, 14 of patients, 9.85%) had a median OS of 27 months (95% CI: 7–NA), while 25–60% partial responders (79 of patients, 55.63%) had a significantly shorter median OS of 6.5 months (95% CI: 6–14). Patients with poor response (<25% response, 27 of patients, 19.01%) exhibited the worst prognosis, with a median OS of only 3 months (95% CI: 1–3). Pairwise comparisons showed that OS was significantly lower in partial and poor responders compared to both complete responders (p < 0.001) and 70–90% responders (p = 0.013 and p = 0.002, respectively).

**Fig 4:**
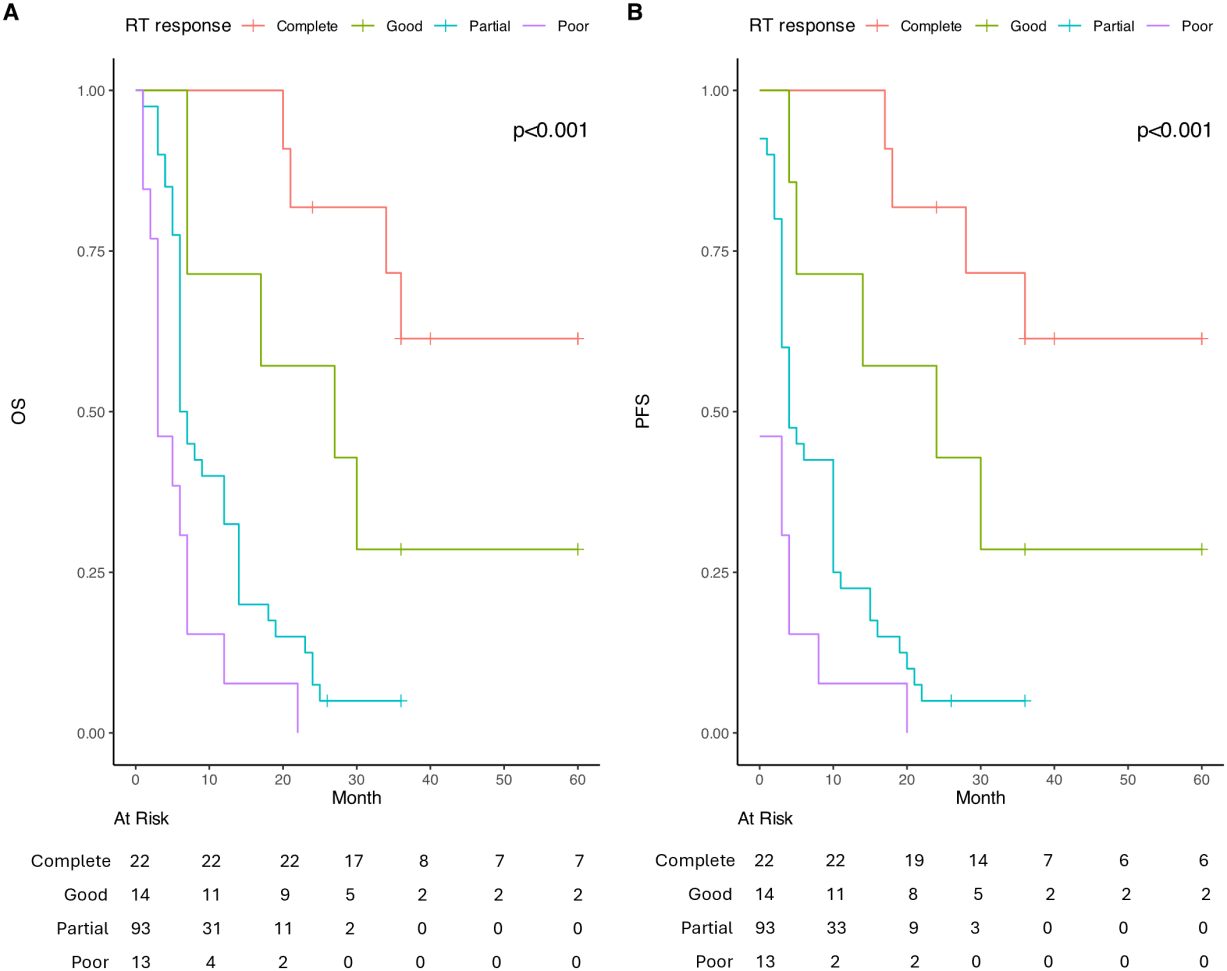
Kaplan Meier curves of OS and PFS according to RT and/or CRT response. A. Comparing OS for different RT rates; B. Comparing PFS for different RT response rates . ROC curve (Raliable Objective Curve) for D-dimer values and ≥1 year OS and PFS are presented in Figs 5 and 6.

A similar trend was observed for PFS, where complete responders had the longest median PFS of 36 months (NA, 95% CI: 28 months-NA), followed by 70–90% responders with 24 months (95% CI: 5–NA). In contrast, 25–60% partial responders had a median PFS of 4 months (95% CI: 3–10), and poor responders had the shortest PFS at 0.5 months (95% CI: 0–3). The differences between groups were statistically significant, with both partial and poor responders showing significantly lower PFS than complete (p < 0.001) and good responders (p = 0.017 and p = 0.002, respectively) (Fig 4).

**Fig 5:**
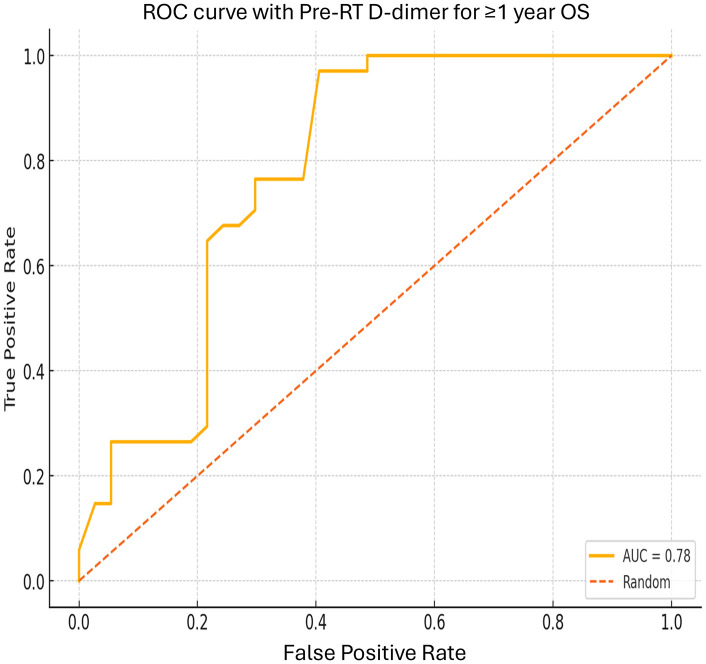
ROC curve according to their D-dimer values in patients with OS ≥ 1 year and those with OS < 1 year. p < 0.001 (95% CI: 6-24). In this study, ROC curve analysis was performed for patients with LC who survived  < 12 months and those who survived  ≥ 12 months, to evaluate OS, PFS, and D-dimer cut-off values.

**Fig 6:**
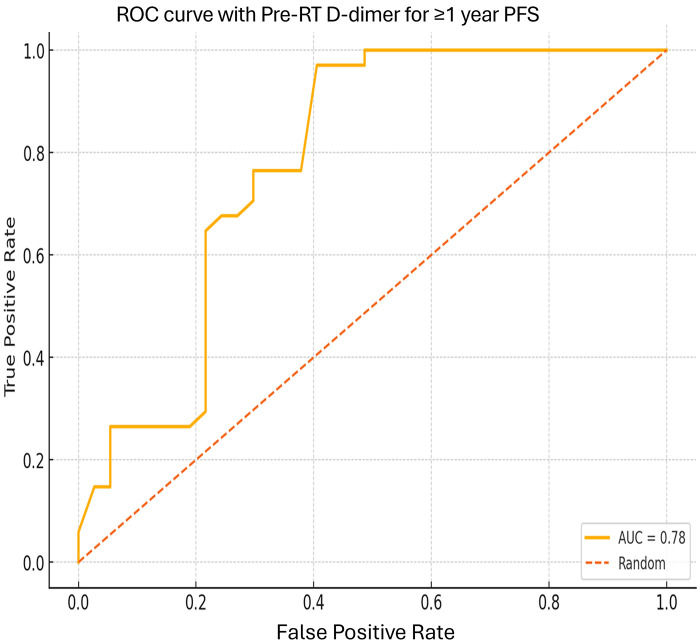
ROC curve according to their D-dimer values in patients with PFS ≥ 1 year and those with PFS < 1 year. p < 0.001 (95% CI: 4-21).

### Univariate and multivariable survival analyses

In univariate analysis for OS, several factors emerged as significant prognostic indicators: stage IV disease (HR = 2.67, 95% CI: 1.35–5.29, p = 0.007), ECOG performance status >2 (HR = 3.06, 95% CI: 1.58–5.91, p < 0.001), total RT dose (HR = 0.96, 95% CI: 0.95–0.98, p < 0.001), receipt of CT (HR = 0.50, 95% CI: 0.29–0.86, p = 0.013), and elevated pre-RT D-dimer levels across all cut-off values (>0.3 µg/mL: HR = 2.63, p = 0.017; > 0.5 µg/mL: HR = 3.62, p < 0.001; > 0.65 µg/mL: HR = 3.40, p < 0.001) (Table 5).

**Table 5:** Univariate and multivariable analyses for OS.

Variable	Univariate		Multivariable	
	HR (95% CI)1	p-value	HR (95% CI)1	p-value
Age	1.00 (0.98–1.03)	0.783		
Male sex	0.70 (0.34–1.42)	0.319		
Pathology, SCLC/ NSCLC	1.18 (0.63–2.23)	0.604		
Stage I-II-III/ IV	2.67 (1.35–5.29)	**0.005**	0.52 (0.16–1.63)	0.260
ECOG ≤ 2/ > 2	3.06 (1.58–5.91)	**<0.001**	2.85 (1.02–7.95)	**0.045**
Total RT dose, ≥ 40–60/ < 40Gy	0.96 (0.95–0.98)	**<0.00**1	0.97 (0.95–1.00)	**0.024**
CT received/ not received	0.50 (0.29–0.86)	**0.01**	0.57 (0.32–1.01)	0.054
Pre-RT D-dimer≤0.3/ > 0.3 µg/mL	2.63 (1.19–5.84)	**0.017**	3.49 (1.48–8.22)	**0.004**
Pre-RT D-dimer ≤0.5/ > 0.5 µg/mL	3.62 (1.88–7.00)	**<0.001**	3.52 (2.08–9.43)	**0.002**
Pre-RT D-dimer ≤0.65/ > 0.65 µg/mL	3.40 (1.83–6.33)	**<0.001**	4.13 (1.48–10.22)	**0.001**
RT response CR/ Others	0.93 (0.85–0.99)	**<0.001**	0.94 (0.93–1)	**0.04**

^1^HR = Hazard ratio, CI = Confidence interval.

Note: Significant p-values are shown in bold.

The subsequent multivariable analysis identified three independent prognostic factors: ECOG performance status >2 (HR = 2.85, 95% CI: 1.02–7.95, p = 0.045), total RT dose (HR = 0.97, 95% CI: 0.95–1.00, p = 0.024), and pre-RT D-dimer >0.3 µg/mL (HR = 3.49, 95% CI: 1.48–8.22, p = 0.004). Among the different D-dimer cut-off values tested in separate multivariable models, the 0.3 µg/mL threshold demonstrated the best model fit. The analysis of PFS yielded comparable results, reinforcing the prognostic significance of these factors (Table 6).

**Table 6:** Univariate and multivariable analyses for progression free survival.

Parameters	Univariate		Multivariate	
Variable	HR (95% CI)1	p-value	HR (95% CI)1	p-value
Age	1.00 (0.98–1.03)	0.798		
Male sex	0.72 (0.35–1.48)	0.378		
SCC/ NSCLC type	1.06 (0.56–2.00)	0.857		
Stage I-II-III/ IV disease	2.54 (1.29–5.00)	**0.007**	0.45 (0.15–1.41)	0.170
ECOG ≤ 2/ > 2	3.00 (1.55–5.80)	**0.001**	2.89 (1.05–7.99)	**0.041**
Total RT dose, ≥ 40–60/ < 40 Gy	0.96 (0.95–0.98)	**<0.001**	0.97 (0.94–0.99)	**0.015**
CT received/ not received	0.53 (0.31–0.92)	**0.023**	0.62 (0.35–1.10)	**0.102**
Pre-RT D-dimer ≤0.3/ > 0.3	2.67 (1.20–5.91)	**0.016**	3.59 (1.53–8.46)	**0.003**
Pre-RT D-dimer ≤0.5/ > 0.5	3.60 (1.88–6.89)	**<0.001**	3.32 (2.08–9.35)	**0.002**
Pre-RT D-dimer ≤0.5/ > 0.65	3.40 (1.84–6.29)	**<0.001**	4.12 (1.46–10.01)	**0.001**
RT response CR/ Other	0.95 (0.92–0.98)	**<0.001**	0.93 (0.91–1)	**0.04**

^1^HR = Hazard ratio, CI = Confidence interval

**Fig 7:**
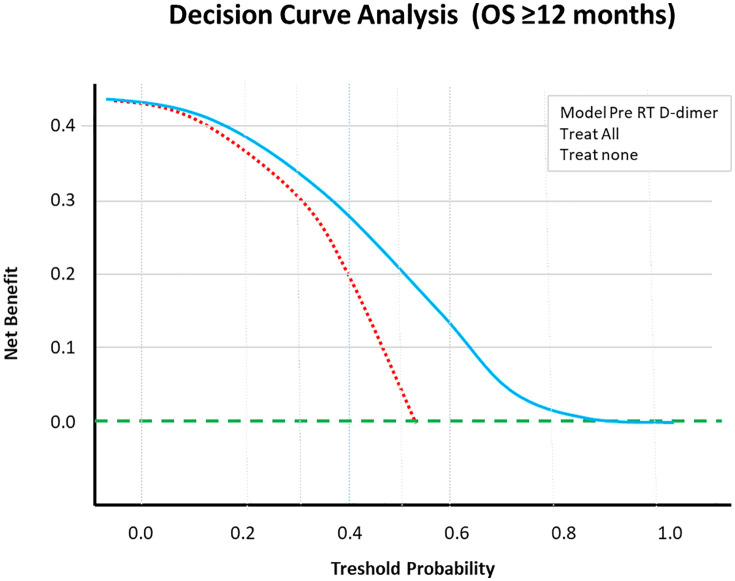
Decision curve analysis according to t D-dimer values in patients with OS ≥ 1 year and those with OS < 1 year. Decision curve analysis was created to enhance ROC analysis and other analyses. D-dimer levels were found to be an important prognostic factor for survival (Fig 7).

### Patient following and response evaluating

Patients were followed up at 1–2 month intervals in the first year for stage III-IV patients, at 2–3 month intervals for others, and at 3–5 month intervals in the second year. During follow-up, computed tomography was performed once every 3 months and PET CT was performed once every 6 months. The median follow-up period was 18 months, (range: 1–60 months).

Response rates were assessed using thoracic Computed Tomography performed within the first 2 months after treatment. In patients with brain metastases brain MRI was conducted was PET CT was performed 3–5 months later.

Response Evaluation Criteria in Solid Tumors (RECIST v1.1 [IV, A]) was used in response evaluation [17].

### RT related toxicity

RT related toxicity was assessed with Common Terminology Criteria for Adverse Events (CTCAE) Version 6.0 [18]. Grade 2 hemathologic toxicity was observed in 83 (58.45%) patients. Grade 3 hematologic toxicity was observed in 14 (9.85%) patients. Grade 3 leg edema and disseminated intravascular coagulopathy were occurred in 6 (4.22%) patients with D-dimer values above 3 during pelvic and lumbar RT, in patients who received total 20 Gy (5x4 Gy).

## Discussion

LC remains a significant public health challenge globally, with NSCLC accounting for the majority of the cases. Because late diagnosis of LC often results in poor prognoses, there is an urgent need to identify inexpensive, easily accessible and widely applicable biomarkers and laboratory tests for early diagnosis to improve patient outcomes.. Coagulation abnormalities are frequently observed in cancer and are known to supports the processes associated with tumor angiogenesis, invasion, and metastasis, ultimately leading to an unfavorable prognosis in patients [4,19–23].

The analysis of plasma D-dimer is vital in the recognition of cardiovascular diseases such as venous thromboembolism (VTE), disseminated intravascular coagulation, infectious syndromes, and malign neoplasms [24].

Unfortunately, D-dimer is not used to determine early diagnosis in early-stage cancers today, as it is only considered as a factor associated with thrombophlebitis, inflammation, and tumor volume. Hovewer, even in cases where the volume of neoplasm is small, it may still metastasize by inducing immunologic, biochemical and physiological changes. indebendently of coagulopathy. High D-dimer levels can sometimes be more predictive than the disease stage in showing the prognosis of the cancer [25]. There are many studies in the literature on the prognostic feature of D-dimer in lung, colorectal, breast, ovarian, and in many other types of cancer [11,26–29].

Antoniou D. et al. conducted a a comprehensive evaluation of plasma D-dimer levels in individuals diagnosed with LC across multiple stages, including measurements taken before, during, and after CT intervention. Their results demonstrated that D-dimer concentrations exhibited a significant decline in patients identified as responders (73.7%), whereas a marked elevation was noted in those experiencing disease progression (68.8%). In this way, the found concentrations of D-dimer in plasma might be important for measuring the effectiveness of therapeutic approaches in LC patients [30].

In this study, Pre-RT D-dimer levels demonstrated significant associations with several clinical parameters: patients with stage IV disease (p = 0.007), like other studies [31]. ECOG performance status >2 (p = 0.003) [32], and those who did not survive during follow-up (p = 0.002) had significantly elevated levels [33].

Recent studies have indicated that elevated D-dimer levels correlate significantly with not only advanced tumor stages but also poorer responses to therapeutic interventions such as CT and RT [34].

The relationship between D-dimer levels and treatment response is particularly noteworthy. In our study the median D-dimer levels, particularly between 0.1 to 1 µg/mL showed a slight decrease after RT. Analyses of study show that this findings correlate with response to RT. Median OS and PFS were found to be better in patients with D-dimer values between 0.1 and 1 (17.5 and 15 months). In patients with pre-RT D-dimer value of less than 1 µg/mL indicate that we can obtain a better response to RT. In contrast patients with pre-RT D-dimer values between 1.1 to 2 µg/mL showed a slight increase after RT, while those with pre-RT D-dimer values between 2.1 to 3 and >3 µg/mL exhibited a median increase of 0.4–1.1 µg/mL after RT. These results were also found to be parallel with RT responses, OS, and PFS rates. In patients with D-dimer values >3 µg/mL, median OS and PFS were 3 and 0.5 months, respectively, and no clear benefit of RT was seen in these patients.

Our analysis indicated that changes in D-dimer levels following treatment could provide insights into therapeutic effectiveness. This observation is consistent with prior research suggesting that monitoring D-dimer levels could inform clinicians about disease progression and treatment efficacy in LC patients [7,8].

In thıs study, median D-dimer level was found to be 0.2 µg/mL in patients with complete and good response, 1.5 µg/mL in partial, and 2.1 µg/mL in poor response. For instance, several meta-analyses found that high D-dimer concentrations were associated with decreased OS and PFS, underscoring its role as a critical indicator of prognosis across various clinical settings [34,35].

We evaluated three different D-dimer cut-off values for their prognostic significance. Among the different D-dimer cut-off values tested in separate multivariate models, the 0.3 µg/mL threshold demonstrated the best model fit [5–8,36]. The analysis of OS and PFS yielded comparable results, reinforcing the prognostic significance of these factors.

Thus, integrating D-dimer assessment into routine clinical practice could enhance decision-making processes and ultimately improve patient management in LC care. This suggests that monitoring D-dimer levels could facilitate more personalized treatment strategies, enabling clinicians to identify patients at higher risk for adverse outcomes who might benefit from intensified therapy or supportive care and alternative approaches.

The predictive and prognostic significance of D-dimer in LC, remains a topic of debate. While some studies support its utility as a prognostic marker, others suggest that its role may be limited due to confounding factors such as inflammation and comorbidities [5,6].

In our study, patients with D-dimer values detected ≤0.3 µg/mL had higher survival rates (Median OS, 30 months) likely because they were diagnosed and treated at an earlier stage, despite having advanced or metastatic lung cancer (LC). Despite many negative factors and conditions in this study, median OS in metastatic disease was 12 months. Median survival rates has been reported as 9−11 month with RT in other publications with unknown D-dimer levels. in stage IV LC patients [37]. In this study, while the median OS was 12 months in stage IV LC patients overall, whereas it increased to 16 months those with D-dimer values between 0.1–0.3 µg/mL. This finding also supports that D-dimer values may be a more important prognostic factor independent of stage. The improved survival outcomes observed in this study may be attributable to the early predictive value of D-dimer.

PET CT, CT and MR examinations cannot be performed at every follow-up to patients every due to the high cost of imaging, side effects with intravenous contrast agent, long term side effects of low dose radiation, high patient load on imaging devices, and overall resource limitations.. Therefore, there may be delays in diagnosis even if there is recurrence or progression in patients. Routinely assessing D-dimer values at every check-up may enable the early detection of disease progression or recurrence.

Another important result of this study is that the median OS is 3 months, in patients with D-dimer values of 3 µg/mL and above. Instead of performing treatments with side effects such as RT or CRT, a treatment approach such as follow-up with supportive treatment can be preferred for these patients.

## Conclusion

Our study reinforces the importance of D-dimer as a potential biomarker in LC patients. Elevated D-dimer levels are associated with advanced disease stages and poorer prognoses, highlighting the need for further research to establish its clinical utility. Future studies should focus on elucidating the underlying mechanisms linking D-dimer levels to tumor biology and exploring its role in guiding treatment strategies.

To our knowledge, no other studies have results on the relaitonship between RT response and D-dimer in LC patients. This study provides a comprehensive analysis on this topic. The D-dimer cut-off analysis in this study is of a nature that may help inform and advance the existing literature. Our finding also emphasize that D-dimer value may serve as an independent prognostic factor beyond traditional staging. However, more comprehensive randomized studies are needed to support its broader implementation in the follow-up of both in early and advanced stage LC patients.
